# Application of Differential Entropy in Characterizing the Deformation Inhomogeneity and Life Prediction of Low-Cycle Fatigue of Metals

**DOI:** 10.3390/ma11101917

**Published:** 2018-10-09

**Authors:** Mu-Hang Zhang, Xiao-Hong Shen, Lei He, Ke-Shi Zhang

**Affiliations:** 1 Key Laboratory of Ocean Acoustics and Sensing, School of Marine Science and Technology, Northwestern Polytechnical University, Xi’an 710072, China; zhangmuhangl@gmail.com (M.-H.Z.); xhshen@nwpu.edu.cn (X.-H.S.); heleimail@gmail.com (L.H.); 2 Key Laboratory of Disaster Prevention and Structural Safety, College of Civil and Architectural Engineering, Guangxi University, Nanning 530004, China

**Keywords:** deformation inhomogeneity, crystal plasticity, differential entropy, low-cycle fatigue, life prediction, polycrystalline metal

## Abstract

The relation between deformation inhomogeneity and low-cycle-fatigue failure of T2 pure copper and the nickel-based superalloy GH4169 under symmetric tension-compression cyclic strain loading is investigated by using a polycrystal representative volume element (RVE) as the material model. The anisotropic behavior of grains and the strain fields are calculated by crystal plasticity, taking the Bauschinger effect into account to track the process of strain cycles of metals, and the Shannon’s differential entropies of both distributions of the strain in the loading direction and the first principal strain are employed at the tension peak of the cycles as measuring parameters of strain inhomogeneity. Both parameters are found to increase in value with increments in the number of cycles and they have critical values for predicting the material’s fatigue failure. Compared to the fatigue test data, it is verified that both parameters measured by Shannon’s differential entropies can be used as fatigue indicating parameters (FIPs) to predict the low cycle fatigue life of metal.

## 1. Introduction

Extensive studies have been conducted in computational mechanics, materials science, and physics to investigate the fatigue lifetime problem and the deformation mechanisms of metals subjected to alternating stress and strain. These have improved our understanding of the fatigue resistance and service life of metallic engineering components and structures. However, the established and widely applied estimation methods, formulas and analyses for fatigue life prediction are based on the conclusions drawn from extensive fatigue experiments [1,2]. Among the competing approaches, Basquin’s formula [3] describes the relationship between the stress state and cyclic fatigue life, and the Manson-Coffin formula [4,5] characterizes the relation between plastic strain and cyclic fatigue life. Both formulas, as well as their combination [6], rely on the lifetime measurement gained in cyclic fatigue experiments under the condition of controlled stress or strain, and the parameters are numerically fitted to match the measurements. These kinds of empirical methods, which generally depend on measured fatigue life data, are still widely used [7,8,9,10,11,12,13,14]. 

Considerable efforts are, therefore, underway towards establishing an analytical model which is capable of reflecting the evolution of fatigue damage in a material undergoing cyclic loading until final failure. For instance, models based on accumulated plastic strain or dissipation energy describe the degree of material damage at the macro-scale [15,16,17,18,19,20], but since they lack any interpretation of the damage mechanism, they still require calibration of model parameters by a series of fatigue experiments, similar to the formulas and models by Basquin [3], Manson-Coffin [4,5] or Morrow [6]. 

Considering that fatigue damage formation and evolution take place at tiny areas, some analytical models [21,22,23,24,25,26,27,28,29] introduce continuum damage or crystal plasticity to describe these processes at the meso and micro level [30,31,32,33,34,35,36,37]. These studies mainly give credit to accumulating plastic strain or energy as sources of damage evolution. The basic assumptions made with respect to the constitutive behavior are significantly different in different studies. The plasticity analysis of crystals takes into account the anisotropy and inhomogeneity of the mesoscopic deformation in polycrystals, whereas models of continuum damage usually assume that the material is isotropic and homogeneous. 

According to the dissipative character of plastic deformation, the cumulative plastic strain or energy are related to the thermodynamic entropy [38]. Considering this relationship, several authors [39,40,41,42,43,44,45] have used this physical quantity as a variable describing fatigue damage. Entropy generation generally results from mechanical dissipation due to plastic deformation, non-recoverable energy due to plastic hardening and damage, and thermal dissipation due to heat conduction. Prevailingly, only plastic work is considered by most authors [40,41,42,43], again. According to Boltzmann [46], thermodynamic entropy can be understood as a representation of disorder, and this disorder parameter may also be employed for characterizing damage evolution [39,45]. Shannon introduced the entropy concept to communication processes to study the amount of information in a transmitted message [47,48]. The definition of the information entropy is, however, quite general and is mathematically equivalent to the Boltzmann entropy. It has, therefore, also found applications for probabilistic fatigue damage prognoses [49,50].

The perception that fatigue damage occurs in regions of very small size gives reason to the relevance of the inhomogeneous micro- or meso-structure of materials. Although it is generally understood that this inhomogeneity causes nonuniform deformation, more systematic research on the role of cyclic loading for local deformation is still needed [35,51,52]. Zhang et al. [53,54] performed crystal plasticity simulations of polycrystals modeled by representative volume elements (RVE) obtained by Voronoi tessellation. They investigated pure copper T2 and the nickel-based superalloy GH4169 under cyclic loading and focused on the evolution of inhomogeneous deformation with an increasing number of cycles. The number of cycles before fatigue failure of these materials is very different, and may be very large when the loading strain amplitude is small, but no matter how large or small the strain amplitude, the increasingly inhomogeneous plastic deformation at the grain level resulting from cyclic loading is very similar. 

The aim of performing numerical analyses of cyclic plastic deformations is to find an appropriate fatigue-indicating parameter (FIP) which correlates with the evolution of damage during the cycles. The study of the fatigue process and FIPs at the micro or meso scale has to account for the inhomogeneous deformation resulting from material heterogeneity. There are multiple factors that trigger inhomogeneous deformation, such as the anisotropy and random orientations of grains, grain boundaries, different phases, precipitations, inclusions and voids, etc. The complexity of the model has to be diminished, however, to reduce the computation time. A simplified model of the material microstructure has been proposed [53,54] considering the metal as a polycrystalline aggregate, the grain boundaries as geometric interface of zero thickness between adjacent grains, and the grains as single crystals, while inclusions and holes are not taken into account. The non-uniform deformation reproduced by this model is mainly dependent on anisotropy and the random orientation of the grains and the plastic deformation by crystal-slip leading to the change of the principal strain directions. The increasing inhomogeneity of deformation in the RVE during cyclic loading which results from changes of the material micro-structure can be characterized by means of statistical analyses of the strain distribution. These investigations brought about the approach of using the statistical standard deviation of the longitudinal strain or the statistical mean of the first principal strain as a FIP to predict fatigue lifetime. The disorder can also be characterized by Shannon entropy [47,48], which was proposed for a quantitative measurement of information and which can be calculated from the distribution of statistical variables. Thus, the change of deformation inhomogeneity of a polycrystalline metal with loading cycles can be represented by the numerical change of entropy, and this change is related to the microstructure evolution of the metal. Thereby, we hope to further understand if it can be adopted as a FIP to predict fatigue failure of materials. To do this, it is necessary to verify whether the calculation of plastic deformation tracking the cycles up to fatigue failure can be carried out, and the entropy takes a critical value that is independent of the load amplitude within a certain range.

In the present paper, we focus on the low-cycle fatigue of polycrystalline metals under strain-controlled symmetrical tensile-compressive cyclic loading, and investigate T2 pure copper at room temperature and the nickel-based superalloy GH4169 at a temperature of 650 °C. 

The outline of the paper is as follows: (1) the Shannon’s differential entropies of longitudinal strain and first principal strain distributions are calculated by crystal plasticity for a polycrystalline RVE subjected to cyclic loading at different strain amplitudes. (2) Their suitability as FIPs is verified by comparing them with the standard deviation of longitudinal strain and the mean of the first principal strain, and their ability for predicting low-cycle fatigue life is discussed.

## 2. Methodology of Strain Inhomogeneity Analysis at the Grain Level for a Polycrystal under Cyclic Loading

The conventional analysis of a polycrystalline metal considers it as a continuous medium, whereas actually the material has a complex micro-structure. Hence, the deformation field is non-uniform at the grain level and becomes increasingly uneven with increasing numbers of cycles. In the present paper, the polycrystalline structure of the material is taken into account; thus, the inhomogeneous elastic-plastic deformation at the grain level and its evolution with loading cycles can be calculated. To avoid any excess of computation time, information on second phases and other micro-structural details within the grain and at the area of the grain boundaries are not included in the model.

### 2.1. Modeling the Material as Representative Volume Element

**Figure 1:**
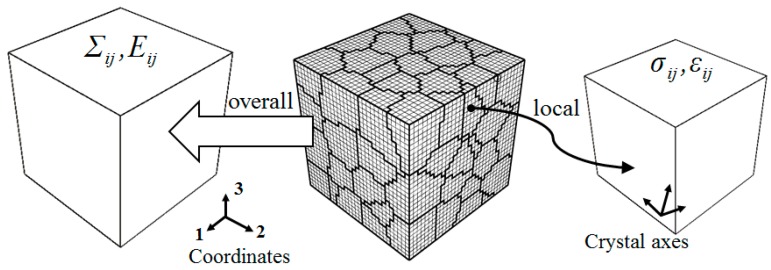
Local and overall stresses and strains of the RVE.

With reference to [53,54], a Voronoi polyhedron aggregation is employed as RVE. The shape, size and crystal orientation of the grains in this model are generated randomly. The plastic deformation of the grains is described by the slip driven by the resolved shear stresses acting at the respective slip systems. The RVE contains 27,000 8-node hexahedral elements and 29,791 nodes, as shown in Figure 1. It is subjected to symmetrical tensile-compressive loading cycles and its surfaces are assumed to remain plane during deformation. 

The macroscopic Cauchy stress tensor Σ and logarithmic strain tensor E are defined as the mean of the local Cauchy stresses σ and logarithmic strains ε over the RVE and calculated as load per unit area and displacements of the surfaces. Crystal plasticity is employed as a constitutive relation between local Cauchy stresses and logarithmic strains within each grain. 

### 2.2. Constitutive Equations of Crystal Plasticity

Plastic slip in the grains is described by the constitutive equations of crystal plasticity proposed by Zhang et al. [53,55], which are based on the fundamental theoretical framework established by Hill and Rice [56], Asaro and Rice [57], Peirce et al. [58] and Needleman et al. [59]. However, the latter did not consider the plastic behavior under cyclic loading.

Extending Hutchinson’s [60] power relation between the shear strain rate, ${\dot{\gamma }}^{\left(\alpha \right)}$, at a slip system, *α*, and the resolved shear stress, $\tau^{\left(\alpha \right)}$, Feng et al. [61] introduced back-stresses and nonlinear kinematic hardening, referring to the Armstrong-Frederick model [62] of the Bauschinger effect,

(1) $${\dot{\gamma }}^{\left(\alpha \right)}={\dot{\gamma }}_{0}\mathrm{sgn}(\tau^{\left(\alpha \right)}-x^{\left(\alpha \right)})\;{\left|\frac{\tau^{\left(\alpha \right)}-x^{\left(\alpha \right)}}{g^{\left(\alpha \right)}}\right|}^{k\prime }\;$$

where ${\dot{\gamma }}_{0}$ is the reference strain rate, being a constant for all slip systems. The resolved back-stress on the *α*-slip system is denoted by $x^{\left(\alpha \right)}$, *k’* is the rate sensitivity parameter and $g^{\left(\alpha \right)}$ defines the domain where the material behaves elastic; its evolution is described as [63]

(2) $$\begin{array}{cc} {\dot{g}}^{\left(\alpha \right)}(\gamma )=\sum_{\beta }^{n}h_{\alpha \beta }(\gamma )\left|{\dot{\gamma }}^{\left(\beta \right)}\right|,\; & \gamma =\int \sum_{\beta }^{n}\left|d\gamma^{\left(\beta \right)}\right| \end{array}\;$$

$h_{\alpha \beta }(\gamma )$ denote the hardening moduli proposed by Hutchinson [64],

(3) $$h_{\alpha \beta }(\gamma )=h(\gamma )[q+(1-q)\delta_{\alpha \beta }]\;$$

*q* is a constant and h(γ) is given according to Chang and Asaro [65] as

(4) $$h(\gamma )=h_{0}{\mathrm{sech}}^{2}\left(\frac{h_{0}\gamma }{\tau_{s}-\tau_{0}}\right)\;$$

where $h_{0}$ is the initial hardening rate, $\tau_{0}$ is the initial critical resolved shear stress, and $\tau_{s}$ is the saturation value. These parameters are regarded as material constants.

The evolution of back-stresses, $x^{\left(\alpha \right)}$, is introduced in [55],

(5) $${\dot{x}}^{\left(\alpha \right)}=a{\dot{\gamma }}^{\left(\alpha \right)}-c\;[1-e_{1}(1-\exp(-e_{2}\gamma ))]\;x^{\left(\alpha \right)}\left|{\dot{\gamma }}^{\left(\alpha \right)}\right|-\lambda x^{\left(\alpha \right)}\;$$

where a, c, $e_{1}$, $e_{2}$ and *λ* are material constants. This formulation includes a strain hardening term, a dynamic recovery term, and a static recovery term. The identification of material constants in Equations (1), (3)–(5) is based on cyclic tests combined with numerical simulations.

The initial unit normal vector of the slip surface of the slip system *α* and the unit vector of the initial direction of the system are denoted as $n^{\left(\alpha \right)}$ and $m^{\left(\alpha \right)}$, respectively. Referring to Hill and Rice [56], Asaro and Rice [57], Peirce et al. [58] and Needleman et al. [59], the Schmid tensor, which establishes the relationship between the shear strain and shear stress in the slip system and its corresponding strain and stress in the Cartesian coordinate system, is given by:

(6) $$P^{(\alpha )*}=\frac{1}{2}(m^{\left(\alpha \right)}{}^{*}n^{\left(\alpha \right)}{}^{*}+n^{\left(\alpha \right)}{}^{*}m^{\left(\alpha \right)}{}^{*})$$

with $m^{(\alpha )*}=F^{*}\cdot m^{\left(\alpha \right)},n^{(\alpha )*}=n^{\left(\alpha \right)}\cdot F^{*-1}$; $F^{*}$ is the elastic term of the deformation gradient tensor, F. The plastic deformation rate tensor can be calculated as

(7) $$D^{p}=\sum_{}^{}P^{(\alpha )*}{\dot{\gamma }}^{\left(\alpha \right)}\;$$

and the resolved shear stress by Schmid’s law,

(8) $$\tau^{\left(\alpha \right)}=P^{(\alpha )*}:\sigma$$

Assuming that the elastic deformations are small, the constitutive relation can be expressed as

(9) $${\dot{\sigma }}^{J}=\overset{<4>}{C}:D^{*}=\overset{<4>}{C}:(D-D^{p})\;$$

where ${\dot{\sigma }}^{J}$ is the Jaumann rate of Cauchy stress,$\overset{<4>}{C}$ is the fourth-order elasticity tensor with respect to the global coordinate axes. During the calculation, the crystal coordinate axes for each grain are rotated along with the changing configuration according to the lattice rotation, since the global coordinate system is fixed.

The incremental change of the Cauchy stress tensor can be calculated as

(10) $${}^{t+\Delta t}\sigma ={{}^{t}\sigma |}_{t+\Delta t}+\Delta \sigma^{J}={{}^{t}\sigma |}_{t+\Delta t}+\overset{<4>}{C}:(\Delta \varepsilon -\Delta \varepsilon^{p})$$

where the increments $\Delta \sigma^{J}$, Δε and $\Delta \varepsilon^{p}$ are determined by integrating the corresponding rates ${\dot{\sigma }}^{J}$, D and $D^{p}$.

For specific numerical implementation as user-supplied subroutine UMAT in the FE code ABAQUS [66], see Zhang et al. [53].

### 2.3. Characterization of the Deformation Inhomogeneity by Statistical Parameters

Due to the randomness of grain shape, size and orientation the strain distribution within the RVE appears to be randomly distributed. As mentioned in the introduction, the deformation of a polycrystalline metal is non-uniform even under uniform macroscopic loading. The distribution of strains at the grain level can be described by statistical parameters like the average of strain components, ${\bar{\varepsilon }}_{ij}=\sum_{k=1}^{n_{\mathrm{RVE}}}{\left(\varepsilon_{ij}\right)}_{k}v_{k}=E_{ij}$, the standard deviation of strain components, ${\hat{\varepsilon }}_{ij}=\sqrt{\sum_{k=1}^{n_{\mathrm{RVE}}}{\left(\varepsilon_{ij}\right)}_{k}^{2}v_{k}-{\bar{\varepsilon }}_{ij}{}^{2}}$, and the maximum of local strains, $\varepsilon_{ij}^{\max}=\underset{n_{\mathrm{RVE}}}{\max}(\varepsilon_{ij})$; where $n_{\mathrm{RVE}}$ is the total number of finite elements in the RVE, $\varepsilon_{ij}$ are the components of the local logarithmic strain tensor, and $v_{k}=\Delta V_{k}/V_{\mathrm{RVE}}$, with $\Delta V_{k}$ being the volume of k-th element and $V_{\mathrm{RVE}}$ the total volume of the RVE. The strain distribution in the polycrystalline RVE changes with the loading cycles and so do the statistical parameters defined above. Zhang et al. [53] proposed to measure the inhomogeneity of micro-strains by the standard deviation, ${\hat{\varepsilon }}_{\left(ll\right)}$, (no summation over subscripts in brackets) of the local longitudinal strain, $\varepsilon_{\left(ll\right)}$, which is the strain in the macroscopic loading direction and is also the direction of macroscopic first principal strain. Its value was found to grow with the number of cycles and can thus be related to the material fatigue failure. Further investigations demonstrated the same behavior for the average, ${\bar{\varepsilon }}_{I}$, and the maximum, $\varepsilon_{I}^{\max}$, of first principal strains [54], which can hence be employed as FIPs and characterize low-cycle fatigue life by comparison with their respective critical values.

This present work attempts to quantify the inhomogeneity of the deformation by using the Shannon entropy calculated from the strain distribution. Entropy initially refers to a probability function characterizing the state of a thermodynamic system and is a measure of its disorder. Shannon and Weaver [48] transferred the concept to quantitative measurement of information and introduced the formula of the information entropy,

(11a) $$H=-\sum_{i}p_{i}\log(p_{i})\;$$

where $p_{i}$ is the probability that an event will occur. The base of the logarithm can be taken as 10, *e* or 2, depending on the object investigated. Here, the value *e* is taken. In Equation (11a), the relative volume fraction of the subdivision region,$v_{i}=\Delta V_{i}/V_{\mathrm{RVE}}$ (with the strain interval $\varepsilon_{i}\leq \varepsilon \leq \varepsilon_{i+1}$) is analogized with $p_{i}$. For a model such as the Voronoi aggregation in which the strain and stress are the continuous variables people can adopt the concept of entropy for continuous distributions which is also referred to as the differential entropy (or continuous entropy) and is given [47],

(11b) $$H=-\int_{-\infty }^{\infty }f_{x}\log f_{x}dx\;$$

where $f_{x}$ denotes the probability density function for a random variable. The differential entropy retains many of the properties of its discrete counterpart, but with some important differences that the details may refer to the literature [67]. In a RVE, the interval of the strain [$\varepsilon_{\min},\;\varepsilon_{\max}$] is divided into *n* subintervals $\Delta \varepsilon_{i}$, i=1,2…,n. In addition, then the integral of the entropy described by Equation (11b) can be numerically calculated according to the following formula:

(12) $$H=-\sum_{i=1}^{n}\left[\frac{p_{\varepsilon_{i}}}{\Delta \varepsilon_{i}}\;\log\;(\frac{p_{\varepsilon_{i}}}{\Delta \varepsilon_{i}})\Delta \varepsilon_{i}\right];\;p_{\varepsilon_{i}}\geq 0,\;for\;\varepsilon_{\min}\leq \varepsilon_{i}\leq \varepsilon_{\max};\;p_{\varepsilon_{i}}=0,\;for\;\mathrm{else}\;\mathrm{case}.\;$$

where, $p_{\varepsilon_{i}}$, is the relative volume fraction, $\Delta V_{i}/V_{\mathrm{RVE}}$, of the region where $\varepsilon_{i}\leq \varepsilon \leq \varepsilon_{i+1}$. For calculation convenience, the interval $\left[\varepsilon_{i},\varepsilon_{i+1}\right]$ in calculation is taken as a constant and $\Delta \varepsilon_{i}=\varepsilon_{i+1}-\varepsilon_{i}=\left(\varepsilon_{\max}-\varepsilon_{\min}\right)/n$; *n* is the division number. It is necessary to point out that the value of the continuous entropy for strain distribution may be negative due to the term $\frac{p_{\varepsilon_{i}}}{\Delta \varepsilon_{i}}$ in Equation (12) being larger than 1, because generally the strain distribution range [$\varepsilon_{\min},\;\varepsilon_{\max}$] is very small. People used to assume entropy to be a positive value; therefore, we use 0.001ε (1000 με) as the unit for measuring the strain interval in Equation (12); thus, the value of $\frac{p_{\varepsilon_{i}}}{\Delta \varepsilon_{i}}$ will be in the range [0, 1], and then the entropy must be positive. This treatment only changes the zero point (reference point), and does not change the entropy difference of different processes.

Thus, Equation (12) is used to describe the inhomogeneity of the distribution in the RVE. The inhomogeneity of the distributions of local longitudinal strain $\varepsilon_{\left(ll\right)}$ and local first principal strain $\varepsilon_{I}$, respectively, are measured by the Shannon’s differential entropies $H_{\varepsilon_{\left(ll\right)}}$ and $H_{\varepsilon_{I}}$,

(13) $$H_{\varepsilon_{\left(ll\right)}}=-\sum_{i}^{n}\left[\frac{p_{\varepsilon_{\left(ll\right)}{}_{i}}}{\Delta \varepsilon_{\left(ll\right)}{}_{i}}\;\log\;(\frac{p_{\varepsilon_{\left(ll\right)}{}_{i}}}{\Delta \varepsilon_{\left(ll\right)}{}_{i}})\Delta \varepsilon_{\left(ll\right)}{}_{i}\right]\;\mathrm{and}\;H_{\varepsilon_{I}}=-\sum_{i}^{n}\left[\frac{p_{\varepsilon_{1}{}_{i}}}{\Delta \varepsilon_{I}{}_{i}}\;\log\;(\frac{p_{\varepsilon_{I}{}_{i}}}{\Delta \varepsilon_{I}{}_{i}})\Delta \varepsilon_{I}{}_{i}\right]$$

where $p_{\varepsilon_{\left(ll\right)}{}_{i}}$ is the relative volume fraction, $\Delta V_{i}/V_{\mathrm{RVE}}$, of the region where $\varepsilon_{(ll)i-1}\leq \varepsilon_{\left(ll\right)}\leq \varepsilon_{(ll)i}$, and $p_{\varepsilon_{1i}}$ is the relative volume fraction of the region where $\varepsilon_{Ii-1}\leq \varepsilon_{I}\leq \varepsilon_{Ii}$, respectively. Equation (13) provides a measure of the deformation inhomogeneity; when the deformation is completely uniform the entropy is zero, generally it is greater than zero, and the more uneven the deformation is, the larger is *H*. The calculated results by using Equation (13) are convergent with the division number, *n*, which is verified by taking a Gaussian distribution function; when the division number is no less than 20 the calculated entropy result will be very close to the saturation value and the error can be ignored.

To verify the rationality of $H_{\varepsilon_{\left(ll\right)}}$ and $H_{\varepsilon_{I}}$ as the parameters characterizing the deformation inhomogeneity and as FIPs, the low-cycle fatigue predictions of the pure copper and the nickel-based superalloy GH4169 are performed by applying both FIPs. In addition, they are further compared with that using the parameters ${\hat{\varepsilon }}_{\left(ll\right)}$ and ${\bar{\varepsilon }}_{I}$, which have been proposed as FIPs in previous publications [53,54].

## 3. Results

For the pure copper and the nickel-based superalloy GH4169, the fatigue experiments are conducted under symmetrical cyclic strain-controlled loading, at room temperature for copper and at a temperature of 650 °C for GH4169. Both materials have the fcc lattice and they are approximated as fcc-equiaxed crystals [53,54]. The effect of grain size is ignored in the material model, and the same geometric partitioning of the RVE (see Figure 1) is used for both. Due to the presence of grain boundaries, inhomogeneous distribution of dispersed phase particles and defects, the very strong strain gradient will lead to a grain size effect. It is difficult to establish a quantitative analysis model based on these complicated factors. Therefore, we regard each grain to be an equivalent crystal with an ideal lattice, and establish a simplified polycrystalline RVE model without size effect, by which the numerical simulation of the whole cycle process can be performed and the difference in grain size for different metals can be ignored.

The chemical compositions of the pure copper T2 and the alloy GH4169 are shown in Table 1 and Table 2, respectively, and the mechanical properties at room temperature for copper and at 650 °C for GH4169 are exhibited in Table 3 and Table 4, respectively.

Based on the finite-element simulation of the RVE of the polycrystals, the parameters of the crystal-plastic model of the materials can be calibrated according to the tested hysteresis-loops. The material model parameters for the pure copper and the cast nickel-base superalloy GH4169 are displayed in Table 5 and Table 6, respectively, according to references [53,54], where the detailed procedure for parameter calibration can be found.

### 3.1. Calculation of Entropy for Fatigue Failure of Pure Copper

**Figure 2:**
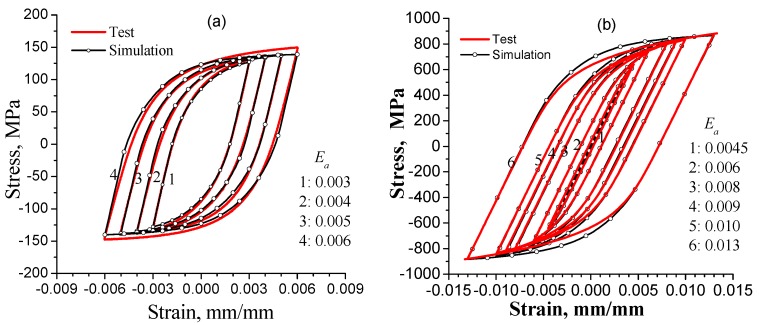
Results for steady and symmetric tensile-compressive hysteresis loops from simulations and experiments: (**a**) for copper T2; (**b**) for superalloy GH4169.

Applying the parameters of Table 5 and Table 6, the stable hysteresis-loops of copper T2 for strain amplitudes 0.003, 0.004, 0.005 and 0.006 and of nickel-based superalloy GH4169 for strain amplitudes 0.0045, 0.006, 0.008, 0.009, 0.01 and 0.013, respectively, as calculated by the RVE simulations are shown in Figure 2. The actually measured steady hysteresis-loops are plotted for comparison. The numerically simulated results accord well with the test data.

#### 3.1.1. Strain Distribution in RVE with Increasing Numbers of Cycles 

**Figure 3:**
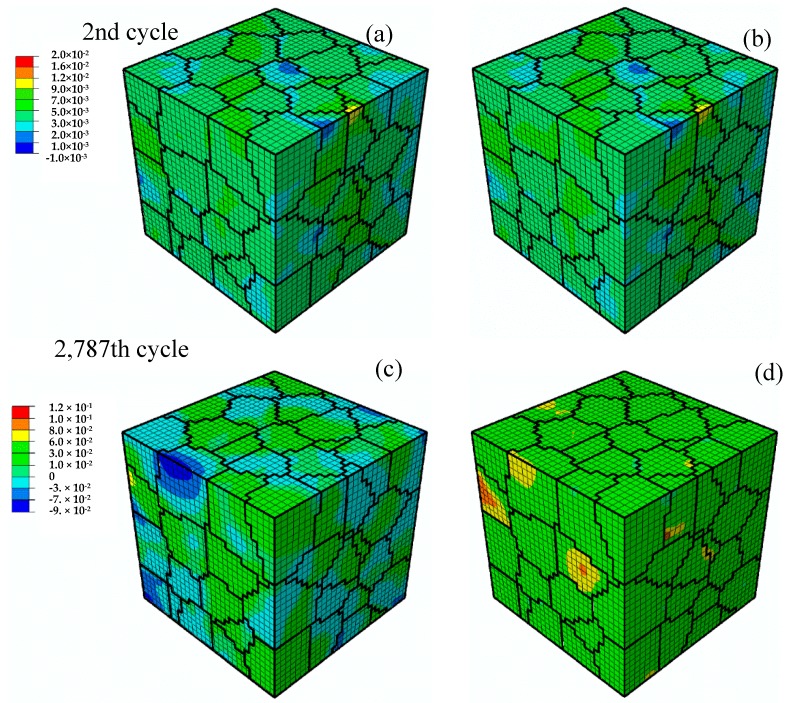
Contours of longitudinal strain, $\varepsilon_{\left(ll\right)}$, and maximum principal strain, $\varepsilon_{I}$, at the second and 2787th tensile peaks of cyclic loading, respectively, at a strain amplitude $E_{a}$ = 0.004: (**a**) $\varepsilon_{\left(ll\right)}$ at 2nd cycle; (**b**)$\varepsilon_{I}$ at 2nd cycle; (**c**)$\varepsilon_{\left(ll\right)}$ at 2787th cycle; (**d**) $\varepsilon_{I}$ at 2787th cycle.

If the factors characterizing the polycrystalline structure—namely, grain orientation and anisotropy properties—are considered in the model, the deformation of the material is not uniform, even under uniform macroscopic loading. Figure 3 displays the contours of the local longitudinal strain, $\varepsilon_{\left(ll\right)}$, i.e., the normal strain along macroscopic loading direction, and the local first principal strain, $\varepsilon_{I}$, at the second and 2787th tensile peak for a strain amplitude $E_{a}$= 0.004. It can be seen from Figure 3a,c that the distribution of the longitudinal strains at the 2787th cycle is much more uneven than that at the second cycle, which implies that the difference of maximum and minimum values in the RVE has become much larger. The same holds for the first principal strain, see Figure 3b,d. 

**Figure 4:**
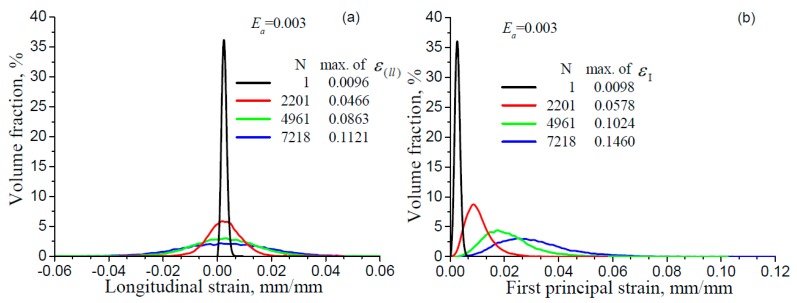

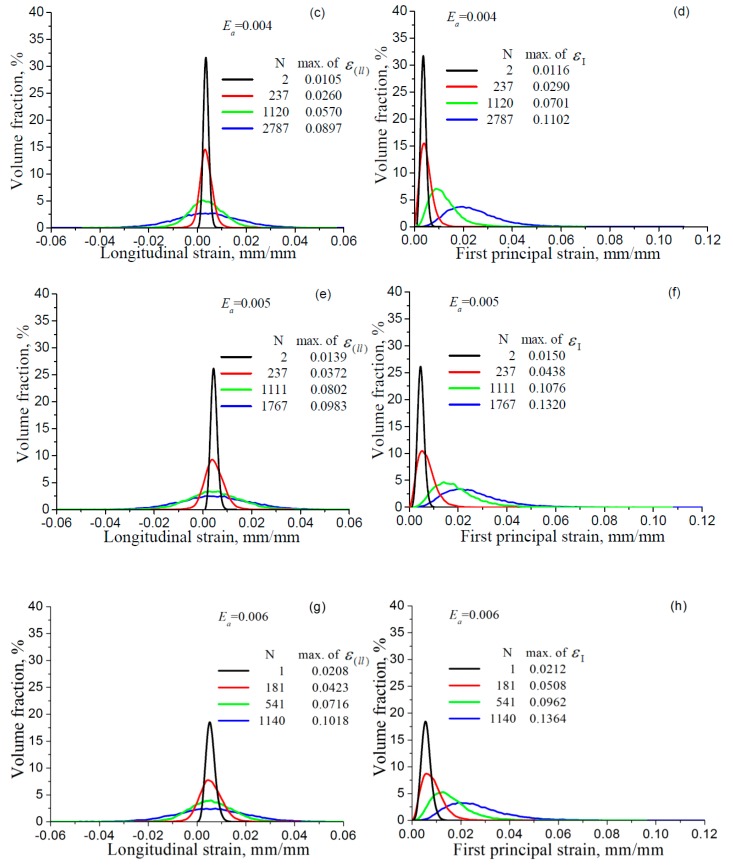
Distribution of the longitudinal strain $\varepsilon_{\left(ll\right)}$ (**a**,**c**,**e**,**g**) and the first principal strain $\varepsilon_{1}$(**b**,**d**,**f**,**h**) in pure copper at the tensile peaks of different cycles for various strain amplitudes.

It needs to be pointed out that the strain contours are differently scaled and the plots cannot clearly visualize the changes from the 2nd to 2787th cycle. A quantitative evaluation of the strain distribution in the RVE and its change with cyclic loading is presented in Figure 4, showing the volume fractions of both the longitudinal strain and the first principal strain at the respective tensile peaks for increasing numbers of cycles number for different strain amplitudes ($E_{a}$ = 0.003, 0.004, 0.005 and 0.006). The variation of strains in the RVE becomes larger and larger and the non-zero range expands with increasing number of cycles.

#### 3.1.2. Predicting Low-Cycle Fatigue Failure by Entropy

**Figure 5:**
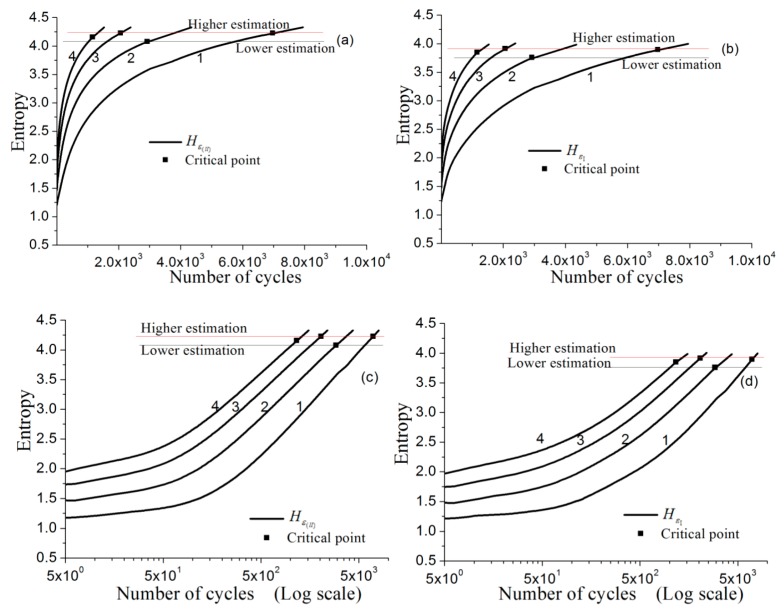
Entropy curves and the corresponding critical value determination for copper T2: (**a**) $H_{\varepsilon_{\left(ll\right)}}(E_{a},N)$, horizontal axis in linear scale; (**b**) $H_{\varepsilon_{I}}(E_{a},N)$, horizontal axis in linear scale; (**c**) $H_{\varepsilon_{\left(ll\right)}}(E_{a},N)$, horizontal axis in logarithmic scale; (**d**) $H_{\varepsilon_{I}}(E_{a},N)$, horizontal axis in logarithmic scale.

By applying Equation (12), we calculate and plot the entropy curves, $H_{\varepsilon_{\left(ll\right)}}(E_{a},N)$ and $H_{\varepsilon_{1}}(E_{a},N)$, as shown in Figure 5. The horizontal axes are linearly scaled in Figure 5a,b, and logarithmically scaled in Figure 5c,d. The information entropies of longitudinal strain and the first principal strain distributions always increase with the number of cycles. The greater the strain amplitude, the faster the entropy values increase. On the logarithmically scaled x-axes, the *H*(*N*) curves tend to become straight with similar asymptotic slopes for high numbers of cycles. 

According to [53], based on the results of symmetric tensile-compressive cyclic experiments and respective calculations of pure copper, the statistical standard deviation of longitudinal strain over the RVE can be applied as a FIP for the fatigue life assessment of a polycrystalline material. Likewise, the results for a GH4169 nickel-based superalloy verify that the statistical standard deviation of the longitudinal strain and the statistical mean of the first principal strain can also be used as FIPs [54]. The suitability of a parameter to be used as a FIP is proved based on its ability to reach a critical value that indicates the occurrences of fatigue failure. In the following, the information entropy of the longitudinal strain and the first principal strain distribution will be examined in terms of whether they are suitable as FIPs. 

The critical entropy values resulting from $H_{\varepsilon_{\left(ll\right)}f}≅H_{\varepsilon_{\left(ll\right)}}(E_{a},N_{f})$ and $H_{\varepsilon_{1}f}≅H_{\varepsilon_{1}}(E_{a},N_{f})$, $N_{f}$ is the fatigue-life cycle number at the specific strain amplitude $E_{a}$, and can be determined from Figure 5. For the respective strain amplitude, the average measured critical values with respect to ${\bar{N}}_{f}$ (see Table 7 column 3) and the upper and lower values with respect to corresponding $N_{f}$ (see Table 7 column 2), obtained from the curves, are listed in Table 7 columns 4 and 5. To prove their rationality, we need to verify whether the critical entropy value determined at any strain amplitude can be used to reasonably predict the fatigue failure at another strain amplitude.

**Figure 6:**
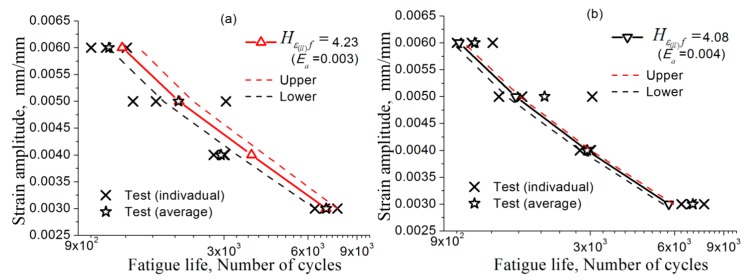

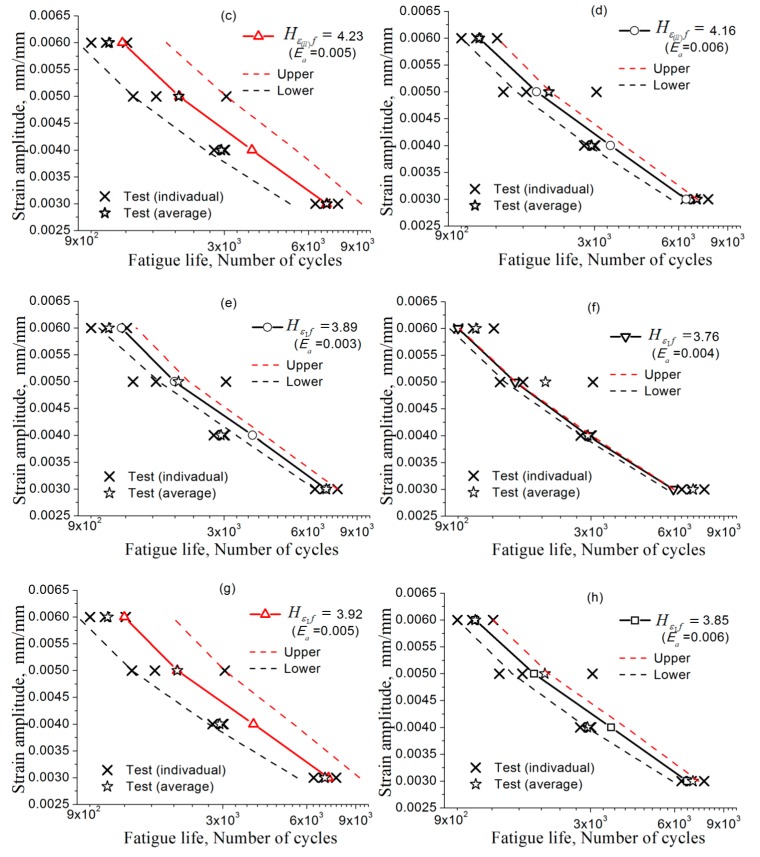
Predicted fatigue-life curves$E_{a}\sim N_{f}$ with the upper and lower estimations based on: (**a**–**d**) $H_{\varepsilon_{\left(ll\right)}f}$ and (**b**–**h**) $H_{\varepsilon_{I}f}$. The test data in this figure are cited from literature [53] (see Table 7).

Taking a critical value in Table 7 column 4 or 5 at any strain amplitude, one can get the intersects from the curves at different strain amplitudes in Figure 5 and get the corresponding fatigue-failure cycles, that is, a fatigue life prediction ($E_{a}\sim N_{f}$) based on the test at single strain amplitude. In the same way, the upper and lower prediction can also be obtained by using the corresponding upper and lower values in Table 7 column 4 or 5. All the results using the present method using the critical value determined from the tests of different strain amplitudes to predict the fatigue lives of the material are listed in Table 8. In addition, the corresponding curves are showed in Figure 6.

The predictions based on $H_{\varepsilon_{\left(ll\right)}f}$ and $H_{\varepsilon_{1}f}$ are proved to be rational in Table 8 and Figure 6, since no matter the average critical value at which the strain amplitude test is taken, the prediction of the fatigue lives for other strain amplitudes are in agreement with the tests. Also, the scattered feature of the tested fatigue life can be illustrated by descriptions of the upper and lower curves in Figure 6. However, the tests are not enough to describe the scattered feature of the fatigue lives; in order to improve the predictions, more tests at the specified strain amplitude are needed for determining the FIP critical value, as well as the upper and lower bounds, more accurately.

**Figure 7:**
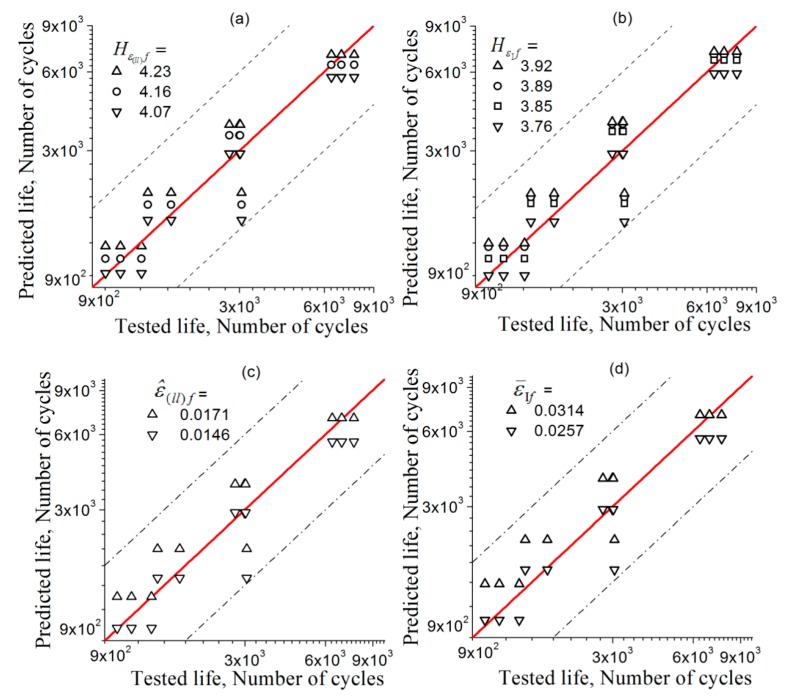
Error assessment between the fatigue lives predicted by various FIPs and the fatigue life tests, the predictions respectively based on: (**a**) $H_{\varepsilon_{\left(ll\right)}f}$; (**b**) $H_{\varepsilon_{1}f}$; (**c**) ${\hat{\varepsilon }}_{(ll)f}$; and (**d**) ${\bar{\varepsilon }}_{If}$.

The error between the predicted and the experimental fatigue lives is shown in Figure 7, including predictions based on standard deviation and mean values of longitudinal and principal strains, ${\hat{\varepsilon }}_{\left(ll\right)}$, and ${\bar{\varepsilon }}_{I}$, data treated according to [53]. The horizontal axis and the vertical axis indicate the actually measured and the predicted life, respectively. The red solid line represents the ideal prediction, and the area between the two dashed lines is the interval of a factor of 2. The error of the prediction results is regarded as acceptable if the data points fall within the double factor region. Hence, Figure 7 shows that using parameters $H_{\varepsilon_{\left(ll\right)}f}$, $H_{\varepsilon_{I}f}$, ${\hat{\varepsilon }}_{(ll)f}$, and ${\bar{\varepsilon }}_{If}$ for the strain amplitudes can give equally reasonable predictions of fatigue lives. Therefore, it is proved possib0le for this method to predict the LCF life of a metal using the present method with its FIP determined based on the test at one strain amplitude. 

### 3.2. Prediction of Fatigue Failure of a Nickel-Based Superalloy by Entropy

Although they both possess FCC lattices, the nickel-base superalloy GH4169 is highly unlike pure copper T2 in terms of mechanical properties, see the hysteresis loops in Figure 2, and the microstructure of GH4169 is far more complex than that of copper T2. By using the same method for analyzing the fatigue-life, we hope to further verify the suitability of Shannon’s differential entropy as FIP not only for pure materials like copper, but also for alloy materials.

#### 3.2.1. Entropy Increase with Cyclic Deformation of GH4169

**Figure 8:**
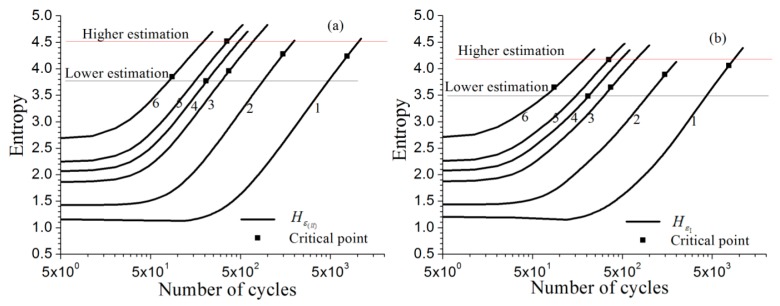
Entropy curves and the critical value determination for the material GH4169 (horizontal axis in log scale): (**a**) $H_{\varepsilon_{\left(ll\right)}}(E_{a},N)$; (**b**) $H_{\varepsilon_{1}}(E_{a},N)$.

Corresponding to different strain amplitudes, the entropy curves, $H_{\varepsilon_{\left(ll\right)}}(E_{a},N)$ and $H_{\varepsilon_{1}}(E_{a},N)$, are calculated dependen on the number of load cycles based on the distribution of longitudinal strain and the distribution of the first principal strain as shown in Figure 8a,b. The horizontal axis is logarithmically scaled. Since the numbers of cycles span three powers of ten, display on a linear horizontal axis is no longer feasible. Similar to the previous analysis of pure copper, the entropy curves tend to a linear growth and the slopes are nearly equal.

**Figure 9:**
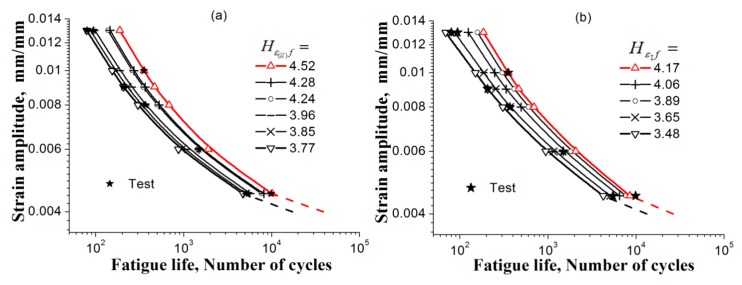
The predicted-life curves $\left(E_{a}\sim N_{f}\right)$ of the material GH4169 at temperature 650 °C based on (**a**) $H_{\varepsilon_{\left(ll\right)}f}$ and (**b**) $H_{\varepsilon_{I}f}$. The test data in these figures are cited from literature [9] (see Table 9).

The curves $H_{\varepsilon_{\left(ll\right)}}(E_{a},N)$ and $H_{\varepsilon_{1}}(E_{a},N)$ for each specified strain amplitude $E_{a}$ are shown in Figure 8a,b; the critical values of the FIPs $H_{\varepsilon_{\left(ll\right)}f}^{}$ and $H_{\varepsilon_{1}f}^{}$ can be picked from each curve by specifying the average of the specific measurement of the fatigue life, these data are listed in Table 9. Then the fatigue lives at different strain amplitudes can be obtained by using any one of these critical values, further drawing a transverse line intersecting with the curves $H_{\varepsilon_{\left(ll\right)}}(E_{a},N)$ or $H_{\varepsilon_{1}}(E_{a},N)$. In addition, abscissa of intersection points on different curve are then taken to represent the estimated fatigue-life value (shown in Table 10) with respect to the specified strain amplitude. The predicted-life curves $\left(E_{a}\sim N_{f}\right)$ based on the FIPs $H_{\varepsilon_{\left(ll\right)}f}^{}$ and $H_{\varepsilon_{1}f}^{}$ are separately displayed in Figure 9a,b. Due only one test for 4 of a total of 6 strain amplitudes having been performed, the upper and lower bounds for each FIP curve cannot be obtained, and the other 2 only had two tests conducted for each. However, the scattered feature of fatigue-life data can also be observed from this figure.

**Figure 10:**
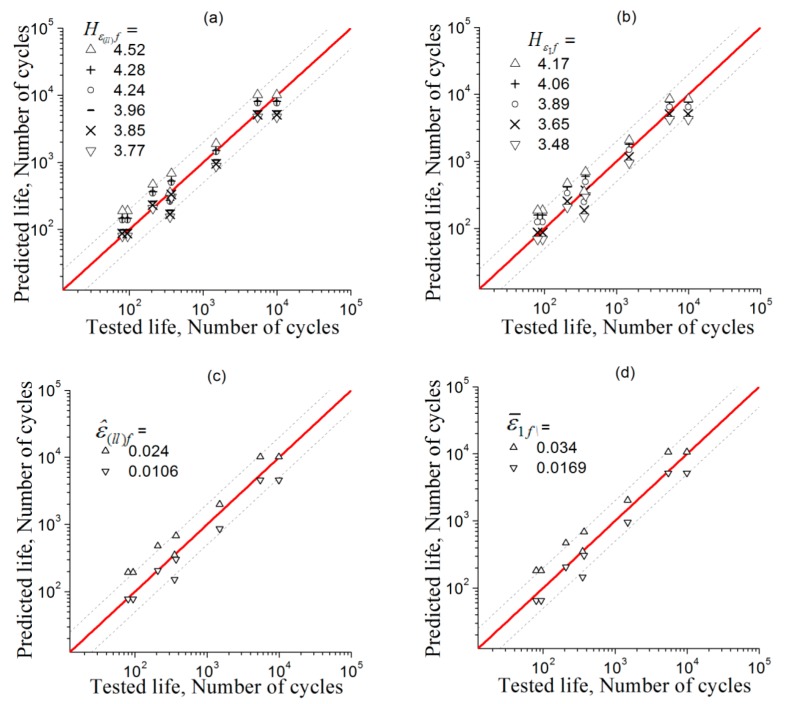
Comparison between predicted fatigue lives with various FIPs and test data of GH4169, the predictions respectively based on: (**a**) $H_{\varepsilon_{\left(ll\right)}f}$; (**b**) $H_{\varepsilon_{1}f}$; (**c**) ${\hat{\varepsilon }}_{(ll)f}$; and (**d**) ${\bar{\varepsilon }}_{If}$.

The fatigue-life predictions by applying FIPs $H_{\varepsilon_{\left(ll\right)}f}$ and $H_{\varepsilon_{I}f}$ together with ${\hat{\varepsilon }}_{(ll)f}$ and , which were suggested in the previous literatures [53,54], are all verified with test fatigue-life data and are shown in Figure 10. For the verification of the FIPs ${\hat{\varepsilon }}_{(ll)f}$ and ${\bar{\varepsilon }}_{1f}$ in this figure, only the predictions based on the highest and lowest critical values are given for the sake of simplicity. From this figure, the predictions are observed to provide reasonable fatigue-life estimations over the range of strain amplitudes under consideration.

#### 3.2.2. The Influence of Model Mesh Size on the Result of Entropy Calculation

The generation of metal polycrystalline RVE models involves many aspects, such as the number of grains, the division of finite element meshes, and randomly generated grain distributions and crystal orientations of grains. Due to random generation, the deformation fields of different polycrystal RVEs obtained by calculation are usually different, and it is necessary to verify whether approximately the same statistical deformation results can be obtained and that they are not sensitive to the random generation of the RVE model. Therefore, whether the model differences influence their description and result with respect to the fatigue law is verified preliminarily by a simple example. In the following, the effect on the entropy calculation caused by the different element size of the model mesh is discussed, which is performed to verify the rationality of the method employing entropy as a FIP. 

**Figure 11:**
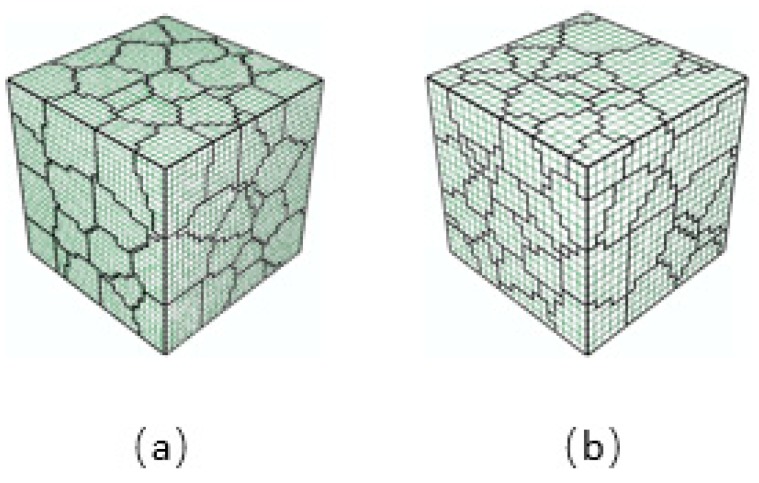
RVE models with different mesh size: (**a**) contains 30 × 30 × 30 (27,000) elements; (**b**) contains 20 × 20 × 20 (8,000) elements.

**Figure 12:**
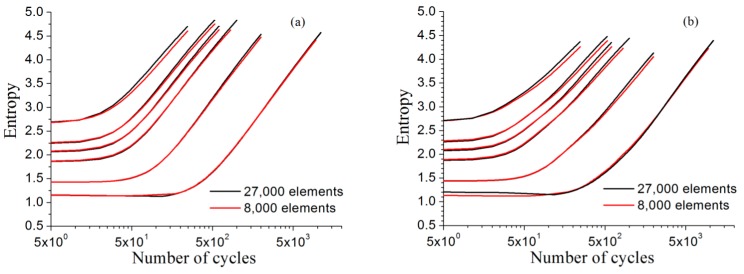
Comparison of the results of entropies calculated from the RVE models with different mesh size for GH4169: (**a**)$H_{\varepsilon_{\left(ll\right)}}(E_{a},N)$; (**b**)$H_{\varepsilon_{1}}(E_{a},N)$.

Considering the RVE model of Section 2.1, the grain number and the orientation for each grain are kept unchanged, but the model is re-divided into a finite element mesh. The new model shown in Figure 11 is the hexahedron RVE divided into 20 × 20 × 20 equal parts along the three directions of length, width, and height, having 8,000 eight-node hexahedral elements and 9,261 nodes. Compared with the original model with 30 × 30 × 30 equal parts, 27,000 elements and 29,791 nodes, the present model, which is much lower in finite element number, will decrease computation time greatly. For the strain amplitudes $E_{a}$ involved in Figure 8, the entropy curves $H_{\varepsilon_{\left(ll\right)}}(E_{a},N)$ and $H_{\varepsilon_{1}}(E_{a},N)$ (red solid lines) using the model with less element number and the corresponding curves (black solid lines) using the original model are plotted together in Figure 12. The curves in the figure show that the difference in the size of the mesh has little effect on the entropy calculation result, which means that the difference of FEM mesh divisions will not cause a noticeable difference in the entropy calculation results.

## 4. Conclusions

In the present paper, the Voronoi polycrystalline RVE is employed, combined with crystal plastic analysis, to simulate the tensile-compressive symmetric strain cycle of the materials T2 pure copper and nickel-base superalloy GH4169. The entropies calculated from of the distribution of the strain in the loading direction and from the distribution of the first principal strain of the materials are employed, respectively, to characterize the inhomogeneous deformation at the tension peak of the cyclic loading and to predict the fatigue lives of materials. Based on the analysis, the following conclusions are obtained:

1. The greater the strain amplitude $E_{a}$, the larger the growing rates with cycles will be for the entropies $H_{\varepsilon_{\left(ll\right)}}(E_{a},N)$ and $H_{\varepsilon_{1}}(E_{a},N)$.
2. Applying the critical values $H_{\varepsilon_{\left(ll\right)}f}$ and $H_{\varepsilon_{1}f}$ to determine the occurrence of metal low-cycle fatigue failure, the predictions are proved rational, and they are also approaches identical to that by applying the critical values ${\hat{\varepsilon }}_{(ll)f}$ and ${\bar{\varepsilon }}_{1f}$ of the FIPs [53,54].
3. Even in the absence of fatigue life data, the $H_{\varepsilon_{\left(ll\right)}}(E_{a},N)$ and $H_{\varepsilon_{1}}(E_{a},N)$ can be obtained by simulation depending only on the material parameters for crystal plasticity. Once the critical values of $H_{\varepsilon_{\left(ll\right)}f}$ and $H_{\varepsilon_{1}f}$ are determined by using the fatigue tests at only single strain amplitude, the fatigue lives can be predicted for other fatigue cycle at different strain amplitudes.
4. The difference of the statistical results of $H_{\varepsilon_{\left(ll\right)}f}$ and $H_{\varepsilon_{1}f}$ from the models with different mesh sizes is very small. This proves that the Shannon’s differential entropy calculation of strain using the present method is not sensitive to the mesh division.

It is necessary to point out that the investigation in the present paper only discusses the prediction of the fatigue lives of a metal under constant strain amplitude loading, and the condition for variable amplitude is not dealt with. In Figure 5 and Figure 8, one can observe that the values of the functions $H_{\varepsilon_{\left(ll\right)}}(E_{a},N)$ and $H_{\varepsilon_{1}}(E_{a},N)$ are larger for the larger strain amplitude loading when the same relative life is consumed for different strain amplitude loading exerted on a material. Therefore, qualitative inferences on fatigue life in variable amplitude condition can be obtained that the life may be longer for such a strain amplitude load sequence from lower to higher, and vice versa. The method may also be considered to apply to multi-axial fatigue issue, but would need to consider more factors, like the influence of tri-axial stress state, how to characterize the non-proportional deformation, etc.

## Figures and Tables

**Table 1:** Chemical composition of the pure copper T2 (wt%).

Cu + Ag	P	Bi	Sb	As	Fe	Ni	Pb	Sn	Zn	Mn	Cd
99.935	0.0416	0.0036	0.001	0.0015	0.0032	0.0023	0.003	0.0019	0.0025	0.0034	0.001

**Table 2:** Chemical composition of the GH4169 superalloy (wt%).

**C**	**Cr**	**Mu**	**Nb + Ta**	**Ni**	**Fe**	**Al**	**Ti**
0.015~0.08	17.0~21.0	2.80~3.80	4.75~5.50	50.0~55.0	Rest	0.30~0.70	0.75~1.15
**Si**	**Mn**	**Co**	**Cu**	**P**	**S**	**B**	
≤0.35	≤0.35	≤1.00	≤0.30	≤0.015	≤0.015	≤0.006	

**Table 3:** Mechanical properties of the pure copper T2.

Young’s Modulus (*E*) GPa	$\mathrm{Yield}\;\mathrm{Stress}\;(\sigma_{0.2})$ MPa	$\mathrm{Tensile}\;\mathrm{Strength}\;(\sigma_{b})$ MPa	$\mathrm{Fracture}\;\mathrm{Strain}\;(\varepsilon_{f})$ mm/mm
108	66.4	297	1.2

**Table 4:** Mechanical properties of the GH4169 superalloy (at 650°C).

Young’s Modulus (*E*) GPa	$\mathrm{Yield}\;\mathrm{Stress}\;(\sigma_{0.2})$ MPa	$\mathrm{Tensile}\;\mathrm{Strength}\;(\sigma_{b})$ MPa	$\mathrm{Fracture}\;\mathrm{Strain}\;(\varepsilon_{f})$ mm/mm
150.5	1230	1090	0.52

**Table 5:** Elastic constants and crystal plasticity parameters of the pure copper T2 [53].

Elastic Constants			Material Parameters of the Crystal Viscoplastic Model											
***C*_11_**	***C*_12_**	***C*_44_**	***τ*_0_**	***τ*_s_**	***h*_0_**	***a***	***c***	*λ*	*e*_1_	*e*_2_	${\dot{\gamma }}_{0}$	*q*		*k′*
GPa	GPa	GPa	MPa	MPa	MPa	MPa	MPa	s^−1^	-		s^−1^			
136.4	98.334	61.074	13.9	30	96	20.6	1.42	0	0.41	5.0	1 × 10^−3^		1	200

**Table 6:** Elastic constants and crystal plasticity parameters of the GH4169 superalloy [54].

Elastic Constants			Material Parameters of the Crystal Viscoplastic Model										
*C*_11_	*C*_12_	*C*_44_	*τ*_0_	*τ*_s_	*h*_0_	*a*	*c*	*λ*	*e*_1_	*e*_2_	${\dot{\gamma }}_{0}$	*q*	*k′*
GPa	GPa	GPa	MPa	MPa	MPa	GPa	GPa	s^−1^	-	-	s^−1^		
206.7	137.98	73.65	260	265	80	35	0.43	0	0	0	1 × 10^−3^	1	150

**Table 7:** Tested low-cycle lives of the pure copper T2 and the critical values of $H_{\varepsilon_{\left(ll\right)}}$ and $H_{\varepsilon_{1}}$.

$E_{a}\;$	$N_{f}$ [53]	${\bar{N}}_{f}\;$	$H_{\varepsilon_{\left(ll\right)}f}\;$	$H_{\varepsilon_{1}f}\;$
			Average/Upper/Lower	Average/Upper/Lower
0.3%	6359/6900/7660	6973	4.23/4.30/4.15	3.89/3.96/3.81
0.4%	2760/2998/3016	2925	4.08/4.10/4.03	3.76/3.762/3.70
0.5%	1417/1714/3050	2060	4.23/4.46/3.99	3.92/4.14/3.69
0.6%	1002/1133/1345	1160	4.16/4.24/4.06	3.85/3.93/3.75

**Table 8:** Prediction of low-cycle lives of the pure copper T2 by applying $H_{\varepsilon_{\left(ll\right)}}$ and$H_{\varepsilon_{1}}$ as FIP.

$E_{a}$	**Estimations Based on** $H_{\varepsilon_{\left(ll\right)}f}$													
	**4.23 (*E_a_* = 0.003)**					**4.08 (*E_a_* = 0.004)**			**4.23 (*E_a_* = 0.005)**			**4.16 (*E_a_* = 0.006)**		
	**Average/Upper/Lower**					**Average/Upper/Lower**			**Average/Upper/Lower**			**Average/Upper/Lower**		
0.003		6973	7660	6359	5728		5951	5469	6973	9297	5149	6385	7006	5636
0.004		3768	4214	3346	2925		3016	2760	3768	5415	2566	3425	3818	2851
0.005		2071	2325	1822	1626		1682	1508	2071	3050	1417	1863	2087	1576
0.006		1295	1482	1153	1021		1061	966	1295	1862	896	1160	1345	1002
$E_{a}$					**Estimations Based on** $H_{\varepsilon_{I}f}^{}$									
		**3.89 (*E_a_* = 0.003)**			**3.76 (*E_a_* = 0.004)**				**3.92 (*E_a_* = 0.005)**			**3.85 (*E_a_* = 0.006)**		
		**Average/Upper/Lower**			**Average/Upper/Lower**				**Average/Upper/Lower**			**Average/Upper/Lower**		
0.003		6973	7660	6359	5925		5960	5603	7182	9279	5543	6655	7321	5889
0.004		3797	4174	3320	2925		3016	2760	3864	5384	2670	3556	4014	2905
0.005		1993	2243	1755	1604		1615	1475	2060	3050	1417	1886	2129	1576
0.006		1286	1454	1067	1001		1013	933	1330	1976	906	1160	1345	1002

**Table 9:** Tested low-cycle fatigue lives of the superalloy GH4169 and critical values of $H_{\varepsilon_{\left(ll\right)}f}^{}$ and $H_{\varepsilon_{1}f}^{}$.

$E_{a}\;$	$N_{f}$ [9]	${\bar{N}}_{f}\;$	$H_{\varepsilon_{\left(ll\right)}f}^{}\;$	$H_{\varepsilon_{1}f}^{}\;$
0.0045	9904/5457	7681	4.24	4.06
0.006	1494	-	4.28	3.89
0.008	370	-	3.96	3.65
0.009	207	-	3.77	3.48
0.010	354	-	4.52	4.17
0.013	80/94	87	3.85	3.65

**Table 10:** Prediction of low-cycle lives of the superalloy GH4169 by applying $H_{\varepsilon_{\left(ll\right)}}$ and $H_{\varepsilon_{1}}$ as FIP.

$E_{a}$	**Estimates Based on** $H_{\varepsilon_{\left(ll\right)}f}$					
	**4.24 (*E_a_* = 0.0045)**	**4.28 (*E_a_* = 0.006)**	**3.96 (*E_a_* = 0.008)**	**3.77 (*E_a_* = 0.009)**	**4.52 (*E_a_* = 0.01)**	**3.85 (*E_a_* = 0.013)**
0.0045	7681	8164	5795	4737	10124	5133
0.006	1440	1494	1086	873	1905	954
0.008	500	527	370	303	683	332
0.009	347	366	260	207	467	230
0.010	260	272	191	154	354	169
0.013	138	146	98	79	188	87
$E_{a}$	**Estimates Based on** $H_{\varepsilon_{1}f}$					
	**4.06 (*E_a_* = 0.0045)**	**3.89 (*E_a_* = 0.006)**	**3.65 (*E_a_* = 0.008)**	**3.48 (*E_a_* = 0.009)**	**4.17 (*E_a_* = 0.01)**	**3.65 (*E_a_* = 0.013)**
0.0045	7681	6497	5097	4250	8548	5097
0.006	1810	1494	1165	946	2067	1165
0.008	601	496	370	308	694	370
0.009	414	336	256	207	466	256
0.010	305	248	187	150	354	187
0.013	159	125	87	69	185	87

## List of Symbols

Σ	Macroscopic Cauchy stress tensor	$\Sigma_{ij}$	Components of macroscopic Cauchy stress tensor
E	Macroscopic logarithmic strain tensor	${Ε}_{ij}$	Components of macroscopic logarithmic strain tensor
σ	Local Cauchy stress tensor	$\sigma_{ij}$	Components of local Cauchy stress tensor
ε	Local logarithmic strain tensor	$\varepsilon_{ij}$	Components of local logarithmic strain tensor
${\dot{\gamma }}^{\left(\alpha \right)}$	Shear strain rate at *α*-slip system	$\tau^{\left(\alpha \right)}$	Resolved shear stress at α-slip system
α, β	Sequence number of slip system	${\dot{\gamma }}_{0}$	Reference strain rate
γ	Accumulated shear slip	$g^{\left(\alpha \right)}$	Critical resolved shear stress at *α*-slip system
${\dot{g}}^{\left(\alpha \right)}$	Evolution rate of $g^{\left(\alpha \right)}$	$x^{\left(\alpha \right)}$	Resolved back-stress on the *α*-slip system
${\dot{x}}^{\left(\alpha \right)}$	Evolution rate of $x^{\left(\alpha \right)}$	*k′*	Rate sensitivity parameter
$\delta_{\alpha \beta }$	Kronecker delta	$h_{\alpha \beta }(\gamma )$	Hardening moduli
$h_{0}$	Initial hardening rate	h(γ)	Hardening function
$\tau_{0}$	Initial critical resolved shear stress	$\tau_{s}$	Saturation value of $g^{\left(\alpha \right)}$
a	Linear hardening coefficient	c	Dynamic recovery coefficient
*λ*	Static recovery coefficient	$e_{1}$, $e_{2}$	Material constants to describe cyclic hardening
$n^{\left(\alpha \right)}$	Unit vectors of the slip plane normal	$m^{\left(\alpha \right)}$	Unit vectors of the slip direction
F	Deformation gradient tensor	$F^{*}$	Elastic part of F
$P^{(\alpha )*}$	Schmid tensor	D	Deformation rate tensor
$D^{*}$	Elastic deformation rate tensor	$D^{p}$	Plastic deformation rate tensor
$\overset{<4>}{C}$	Fourth-order elasticity tensor	${\dot{\sigma }}^{J}$	Jaumann rate of Cauchy stress
${}^{t}\sigma$	Cauchy stress tensor at time *t*	${}^{t+\Delta t}\sigma$	Cauchy stress tensor at time *t+*Δ*t*
${{}^{t}\sigma |}_{t+\Delta t}$	Cauchy stress tensor under the configuration at time *t+*Δ*t*	${\bar{\varepsilon }}_{ij}$	Average of local strain components over the RVE
${\hat{\varepsilon }}_{ij}$	Standard deviation of local strain components over the RVE	$v_{k}$	Relative volume of k-th element
$\Delta V_{k}$	Volume of *k*-th element	$V_{\mathrm{RVE}}$	Total volume of the RVE
$\varepsilon_{\left(ll\right)}$	Normal strain in the macroscopic loading direction	${\bar{\varepsilon }}_{I}$	First principal strain
$p_{\varepsilon_{i}}$	Relative volume fraction of the region where $\varepsilon_{i}\leq \varepsilon \leq \varepsilon_{i+1}$	$\Delta V_{i}$	Volume of the subdivision region with the strain interval $\varepsilon_{i}\leq \varepsilon \leq \varepsilon_{i+1}$
H	Shannon’s information entropy	$H_{\varepsilon_{\left(ll\right)}}$	Shannon’s differential entropy for $\varepsilon_{\left(ll\right)}$
$H_{\varepsilon_{I}}$	Shannon’s differential entropy for $\varepsilon_{I}$		
